# Advising adolescents on the use of psychotropic medication: attitudes among medical and psychology students

**DOI:** 10.1186/1747-597X-2-21

**Published:** 2007-07-12

**Authors:** Michèle Baumann, Elisabeth Spitz

**Affiliations:** 1 Integrative research unit on social and individual development (INSIDE), University of Luxembourg, Walferdange, Luxembourg; 2 Department of Health Psychology, University of Paul Verlaine, Metz, France

## Abstract

**Background:**

There is evidence that medical students are more aware of the benefits of psychotropic treatment than are members of the general public, and that the more knowledge students acquire about psychiatry and pharmacology, the more favorable their attitudes become towards psychotropic drugs and other treatments.

**Objectives:**

This study among students investigates the relationship between certain aspects of personality and attitudes towards advising adolescents with psychosocial problems about the use of psychotropic medication.

**Methods:**

Two groups of healthcare students were recruited from universities in Eastern France. 41 fourth-year medical students (MS) who had completed their psychiatry course, and 76 third-year psychology students (PS) in the faculty of human sciences. Respondents completed a self-administered instrument (20 brief case studies, and a personality inventory) at the end of a lecture. Participation was voluntary and unpaid.

**Results:**

MS would recommend psychotropic drugs in 40% of the 20 cases, PS in 27%. MS who would prescribe psychotropic medication differed in personality profile from PS. MS with a tendency to experience anger and related states such as frustration, and who did not see fulfilling moral obligations as important were more likely to prescribe psychotropic drugs. Also more likely to recommend psychotropic drugs, but for different reasons, were PS who were susceptible to stress but not shy or socially anxious, who showed friendliness but little interest in others, and who lacked distance in their decision-making.

**Conclusion:**

Health promotion is not simply a matter of educating those young people who take psychotropic drugs – health professionals must also question the criteria that inform their decisions. It is as important to investigate the attitudes of the future health professionals (advisers or prescribers) as it is to focus on consumer-related issues.

## Background

Psychotropic medications are widely prescribed by general practitioners in Europe as a whole, and the highest levels of prescription are seen in France [1,2]. Increasing consumption among adolescents is a major public health problem [3], regardless of whether the medication is prescribed or obtained informally (from a family member, for example). Several epidemiological studies have reported increases in their prescription [4,5]. The potential consequences of psychotropic drug use include morbidity, mortality, deleterious effects on quality of life, and the cost of remedial treatments [6,7]. More specifically with regard to adolescent health, psychotropic medication carries a risk of undesirable side-effects, the development of dependency, and delayed initiation of adequate management of the underlying problem. Furthermore, relief of symptoms may result in the patient neglecting to follow appropriate health-related advice or failing to modify dangerous habits. Rapid progress in medicine has reinforced this trend, as have advertising and the greater availability of certain drugs – putting the younger generation at an increasing risk [8].

Providing GPs – and other relevant non-specialist health professionals – with training in psychiatry would be expected to reduce inappropriate administration. According to some authors [9,10], medical students are more likely than members of the general public to be aware of the benefits of psychotropic treatment. One survey [11] found that the general population in Germany has more faith in psychotherapy than in pharmacological approaches, due to concerns about potential dependency. For them, psychotropic medications and drug abuse had comparable negative images. An investigation in the USA reported similar, but even less favorable, attitudes [12]. Other German surveys, and some conducted in Australia, indicate that the general public equate the use of psychotropic medication with having a weak personality[13,14].

A survey carried out among 4545 adolescent patients reported that five different treatment components (individual psychotherapy with the patient, functional therapies, parent-and family-oriented interventions, other environmental interventions and psychotropic medication) were used in various combinations to treat a range of disorders in diverse settings [15]. It found that although psychotherapy has traditionally been the principal treatment for depression in adolescents, there has recently been an increase in the prescription of antidepressants to this age group. Approximately three quarters (79%) of treated adolescents received psychotherapy and more than half (60%) were prescribed psychotropic medications. Antidepressant medications appear to be used far more commonly than would be expected on the basis of published treatment recommendations [16]. A review of the literature concerning "juvenile maladaptive aggression" published in 2006 shows that "therapeutic nihilism" in the treatment of aggressive children and adolescents with conduct problems is no longer warranted. The best evidence for effectiveness is in multifocused psychosocial interventions given early in life to at-risk children and adolescents [17].

Observations of this kind prompted the present authors to consider factors that might influence attitudes towards counseling about the use of psychotropic drugs and their administration. University students completing courses in medicine or psychology were chosen in the light of the recommendation by numerous authors that a two-pronged approach (medication and psychotherapy) can be taken to manage psychosocial problems in adolescents [18].

Because of heavy workloads, improvements in this area are likely to focus on the quality of interactions between young people and health professionals, rather than on increasing the length of consultations. However, the first contact should be sufficiently long to establish a worthwhile relationship, and help close the gap between what we really know about mental health difficulties, and the messages that society gives. It may be that visits of that kind should be given priority status and funding.

Although the international literature contains no investigations about the relationship between prescription of psychotropic medication and professional personality traits, a link is plausible.

The objective here was to obtain answers to the following questions: what advice would students of medicine and psychology give adolescents concerning the use of psychotropic drugs in coping with difficult life events? Is there a statistically significant difference between the two groups? Is it possible to identify aspects of personality that discriminate between students with different attitudes towards advising adolescents with psychosocial problems about the use of psychotropic medication?

## Methods

### Subjects

Two groups of future healthcare professionals studying at French universities were recruited for this cross-sectional survey. Participants ranged in age from 19 to 30 years. The first group comprised 41 fourth-year medical students (**MS**) who had completed their psychiatry course; their average age was 22.6 years (SD = 2.3), and 61% were female. The second group were 76 third-year psychology students (**PS**) of whom 89% were female; the average age was 22.7 years (SD = 1.8) (*t *= 0.39; *p *= 0.69).

### Data collection

Respondents completed a two-part self-administered instrument at the end of a lecture given at each university. Participation was voluntary and unpaid.

The first part of the questionnaire was designed to elicit the attitudes of respondents to the administration of psychotropic drugs to adolescents. It comprised 20 case studies of young people (10 males and 10 females) going through difficult life events. The situations described were reported by general practitioners and psychiatrists [19]. Each case study was accompanied by the following question: '*In your opinion, does this adolescent need psychotropic drugs?'*. The possible answers were "Yes" and "No". The second part of the instrument measured facets of personality using a selection of the 240 assertions in the Costa & McCrae revised NEO Personality Inventory (NEO PI-R) [20]. A panel comprising the steering group plus a sociologist and three psychologists selected 112 assertions exploring the fields of: neuroticism (anxiety, hostility, depression, self-consciousness, impulsiveness, vulnerability to stress), extraversion (warmth), agreeableness (trust, altruism), conscientiousness (competence, dutifulness, deliberation) and openness to experience (feelings, openness), all of which were considered to have a potential effect on attitudes towards advice on taking psychotropic drugs.

Subjects responded to each of the 112 assertions by circling a letter that best described their opinion (in French), as follows: FD (strongly disagree), D (disagree), N (neutral), A (agree), FA (strongly agree).

### Statistical analysis

The chi2 test was used to compare the proportions of each group (MS and PS) with favourable attitudes towards the prescription of psychotropic drugs. Two linear regressions were carried out *(type III Anova tests)*. We have used the computer program SPSS. An 'advising medication score' was calculated for each student by multiplying the number of positive responses to the case studies question (n = 20) by 5, to give a percentage. This was adopted as the dependent variable. The sex of respondents was considered.

## Results

As Table 1 shows, the difference between the two student groups was significant in seven of the 20 cases (35%), but did not reach significance in the majority (65%). No statistically significant difference was observed between female and male students in their responses to the case studies. Men would prescribe psychotropic drugs in 36% of the cases, and women in 30.6 % (*x2 *= 1.33; *df *= 1; *p *= 0.25).

**Table 1:** Frequency of students advising psychotropic medication

**Case descriptions**	**Medical students**	**Psycho students**
**Case 1: Julie**, aged 20, 2^nd ^year French student, living in a hostel for 3 months, goes home every weekend, oldest child in the family with 3 younger brothers, aged 5, 10 and 15. *Health and psychosomatic disorders: *suicide attempt one year ago, many somatic symptoms: headaches, nausea, constipation, digestive disorders, eating disorders, sleep disorders, and anxiety, which has decreased since a loving relationship began.	17.1%	15.8%
**Case 2: Patrick**, aged 19, final year in a secondary school, and lives with his mother, his step-father and his two half brothers. When he was 15, he learned that his foster father was not his biological father. At the moment he is trying to get closer to his father, whom he does not know. The request for a consultation came from the family, and concerns his 'tantrums': he is drunk most of the time and becomes very violent – verbally as well as physically. He can go to extremes, provoking fights or riding his motorbike at 200 kph, and has no memory of anything the next day. His family no longer recognizes him as being himself.	**82.9%**	42.1%***
**Case 3: Nadia**, aged 20, is a final year student in a secondary school: she lives at home with her parents and 2 brothers. In the course of this year, many conflicts have appeared. At school her role as class delegate has caused problems. At home, she complains about a lack of understanding by her parents. At the moment she suffers fromviolent headaches, difficulties in going to sleep and a loss of appetite. She suffers from anxiety, is world weary, and thinks of attempting suicide.	**61.0%**	44.7%*
**Case 4: Philippe**, aged 20, is a 2^nd ^year student in a school of engineering and lives in a student residence. After intellectual burn-out he did not take his exams and now feels discredited and guilty in the face of his parents who finance his studies. At the moment he feels tired, and apathetic. He is riddled with doubts and wonders about his future. He also feels a lack of interest in anything and tends to retreat inside himself.	17.1%	14.5%
**Case 5: Claire**, aged 19, is a 2^nd ^year student reading physics and chemistry. She lives at home with her parents but the family atmosphere has become very tense over recent months. Claire's father is very authoritarian and always wants to control her every action, especially concerning her love life. A few days ago she ran away from home to join a friend. At the moment she complains of intense tiredness, says she is depressed, has doubts and hesitations and is always afraid of upsetting someone or doing something badly.	14.6%	6.6%
**Case 6: Emmanuel**, aged 18, is in year 11 and lives at home. He feels very lonely and would like to have someone to confide in. He says that he suffers from having neither brothers nor sisters. He has started smoking heavily over the past few months, influenced by his friends. He presents motor coordination and verbal expression troubles, which he states would be a result of his intoxication. He says that he is troubled and needs to talk.	31.7%	18.4%
**Case 7: Claudine**, aged 20, is a 2^nd ^year law student living with her father. Since her mother's death 4 years ago she has suffered from bouts of sadness. Her father offers her little emotional support and their relationship is painful to them both. These difficulties create a feeling of frustration and rejection in her.	26.8%	27.6%
**Case 8: Azzedine**, aged 17, is in the 1^st ^year of "*Maths SUP*" (high-flyer studies) and lives in a student hall. Daily violent headaches prevent him from working as he would like. Their sudden and frequent onset makes him anxious all day about a new attack. He feels irritable, bad tempered and tends to avoid his friends more and more.	36.6%	23.7%
**Case 9: Francesca**, aged 20, dropped out during her vocational accounting training. She has been living with her boyfriend for almost a year. Her behavior has changed since she came back from a trip abroad with her father. She is obsessed with her weight and no longer eats. She claims not to feel particularly sad, but immense fatigue limits her actions. She often feels the need to purify herself, sometimes taking up to 4 showers a day.	36.6%	32.9%
**Case 10: Mathieu**, aged 19, is an apprentice garage mechanic, and has been living with his girlfriend for 2 years. He has been suffering from vertigo accompanied by a feeling of suffocation and oppression for some time. Because he is afraid of having an attack in public, he dares go out less and less. He says that he feels emptied of any energy and finds it impossible to take any initiatives.	**63.4%**	32.9%**
Case **11: Natacha**, aged 20, has no training and no income. She lives with a friend after being placed in a welfare department home and then with a foster family. At present she is in major conflict with her friend and is tending to isolate herself more and more from any relationship. Having a gloomy character, she states that she is incapable of making any decision, however small, or taking any action.	14.6%	15.8%
**Case 12: Frédéric**, aged 20, is unemployed and shares a home with a friend. He consults because of a sleep disorder. He goes to sleep easily, but wakes up every morning at 4 a.m. and can't get back to sleep. He is tired during the day with drawn features and a lack of energy. He says that he is not particularly worried about his hunt for a job and would be able to look on the bright side of life if he didn't have the sleep disorder.	**36.6%**	19.7%*
**Case 13: Elodie**, aged 16, is a year 12 student and lives at home. Her mother, who comes with her, states that her daughter gained 10 kgs in a year. She does not each much during meals, but snacks a lot during the day and especially in the evening. She has problems accepting her classmates' scrutiny, feels ill at ease amongst them and has few friends. To make up for her relationship difficulties, she says that she 'throws herself' at her food, as soon as she arrives home. She would like to take appetite suppressors in order to help her stick to her diet.	17.1%	15.8%
**Case 14: Michel**, aged 17, is a final year school student, living at home. His brother died 5 months ago and since then, Michel has shown behavior troubles. He no longer dares cross the road for fear of being knocked down, or go swimming for fear of drowning. He can no longer make plans for the future and states that he sees his life in the short term. He seems very upset and anxious.	85.4%	73.7%
**Case 15: Christelle**, aged 21, is a hairdresser and lives at home with her parents. She is unstable and suffers from repeated emotional breakdowns, the last of which left her pessimistic and likely to avoid situations that could make her suffer. Since this failure, she eats or drinks nothing other than coffee with milk and suffers from insomnia. She says elsewhere that she no longer has any aims in life.	**58.5%**	28.9%**
**Case 16: Kevin**, aged 21, sells tickets in the suburban zone of the Paris underground. His working hours do not allow him to enjoy his day. He claims to be very sociable, but admits to being more and more afraid of crowds. He would like to change environments, but he needs this job to pay his rent. He feels invaded and oppressed and suffers from anxiety.	**41.5%**	15.8%***
**Case 17: Valérie**, aged 19, is working towards a vocational secretarial diploma and lives in a hostel. Her family relationships are quite poor and her social relationships are very flimsy. She feels rejected, is very irritable and has difficulty in accepting frustration. For some time she has been presenting a very destabilizing alternation of overexcitement and bouts of anxiety.	**82.9%**	51.3%***
**Case 18: Franck**, aged 17, is a year 12 student who lives at home with his parents. His sister brings him along because she is worried about her brother's poor school results. The problem started 6 months ago when their father, who is a long distance lorry driver, went to work abroad. The situation is worrying Franck and making him unstable. He feels sad and worried, abandoned and all alone in the world.	12.2%	2.6%
**Case 19: Laëtitia**, aged 20, is a checkout assistant, and lives with a childhood friend. She left her parents' home 2 years ago during a crisis in which she accused her stepfather of sexual abuse. Now she appears introverted, always in the background, and uncommunicative. She seems to be worried and reticent and says she is quite anxious about life in general.	39.0%	31.6%
**Case 20: Jérôme**, aged 20, is an apprentice mason and lives with his parents and younger brother. After a few minutes of silence, he says that his father sleeps all the time and does not do anything in the house. He then admits that his father has problems with alcohol and that he has just come back from drying out treatment. He states that he has too much responsibility at home, and feels that he is the pillar of the family. He feels fragile and disoriented, and says that he no longer has the strength and starts crying.	34.1%	34.2%

**Total (all 20 case studies)**	**40.5%**	27.3%***

* p < 0.05; ** p < 0.01; ***p < 0.001 levels of significance

Overall, mean case-study scores were statistically significantly higher (*x2 *= 4.46; *df *= 1; *p *= 0.035) among MS (40, 5%) than in the group studying psychology (27, 3%). In other words, MS would prescribe psychotropic drugs in 40% of cases, compared with 27% among PS.

MS were more likely than PS to favour psychotropic medications when the case described behaviour that might put the adolescent's life at risk. In two cases, more than 80% of MS, but 40–50% of PS would prescribe psychotropic drugs (2: Patrick; 17: Valérie, both of whom exhibit behaviour putting them at risk, and impulsivity). Mental disorders (tantrums and destabilising overexcitement) are present in these cases.

MS would recommend psychotropic medication significantly more often than would PS. Significant differences between the two groups were marked in five cases: (3: Nadia who presents with depressive affect's 10: Mathieu, who is afraid of panic attacks, 12: Frédéric, who is tired and has sleep disorder, 15: Christelle, who claims to no longer have a goal in life, and 16: Kevin, who suffers from anxiety). They have in common a lack of aims in life, and feelings of oppression.

In just one case more than 73% of both groups would prescribe psychotropic drugs (14: Michel, whose brother died 5 months previously and who showed behavior disorders).

In six other cases, 20–40% of both groups favored psychotropic drugs (6: Emmanuel; 7: Claudine; 8: Azzedine; 9: Francesca; 19: Laetitia; 20: Jérôme). They were all distressed, irritable, and felt rejected by others.

In six cases, fewer than 20% of the MS and PS would prescribe (or recommend) psychotropic drugs (1: Julie; 4: Philippe; 5: Claire; 11: Natacha; 13: Elodie; 18: Franck). The common elements are disinterest, tiredness and, particularly, loss of self-confidence.

Table 2. By retaining the most significant variables, we obtain two separate linear models for the MS and the PS. Parameter estimates are shown in table 2. The models explain 23% (MS) and 21% (PS) of the variance in the proportions advising medication. The predicted means of the percentages advising medication (0.40 [0.37; 0.44], 0.27 [0.24; 0.31], respectively) are essentially identical to the crude percentages, showing that the MS are more likely to advise medication.

**Table 2:** Regression analyses to explain psychotropic drug prescription according to aspects of personality among medical students and psychology students.

	**Parameter**	**[95% CI]**	**df**	**p**
**Medical students**				
(Intercept)	0.507	[0.25; 0.76]	1	0.0003 ***
Anger	0.067	[0.01; 0.12]	1	0.0144 *
Sense of duty	-0.074	[-0.15; 0.00]	1	0.0558

**Psychology students**				
(Intercept)	0.355	[-0.02; 0.73]	1	0.0611
Shyness	-0.094	[-0.18; 0.00]	1	0.0398 *
Vulnerability	0.123	[0.04; 0.21]	1	0.0052 **
Warmth	-0.110	[-0.21; -0.01]	1	0.0373 *
Altruism	0.131	[0.01; 0.25]	1	0.0286 *
Deliberation	-0.065	[-0.13; 0.00]	1	0.0543

* p < 0.05; ** p < 0.01 ; *** p < 0.001 levels of significance of type III Anova tests.

Table 2 shows that MS who favour prescribing psychotropic medication differ in personality profile from the psychology students. MS who have a tendency to experience anger and related states such as frustration and bitterness, and do not consider fulfilling moral obligations as important are more likely to prescribe psychotropic drugs.

PS likely to favour medication tend to be susceptible to stress, and to panic when they are in problematic situations. However, they are not shy or socially anxious, not disturbed by embarrassing social circumstances, often speak and act without considering the consequences and lack distance in their decisions.

## Discussion

The major findings in this study are: first that, overall (considering all the cases) the majority of – respondents (PS+MS) do not tend to prescribe psychotropic medication; second, certain personality traits relate to willingness to prescribe/recommend psychotropic medication.

In all cases, the MS who had been taught psychiatry tended to have more favorable attitudes towards psychotropic medications than did PS (who would never have the opportunity to prescribe them). It is plausible that PS, are less likely to favor medication because they have learned that psychotherapy is a good way to deal with some problems. This assumption is in accord with the results of German studies [10,11].

The most important differences between the answers of the MS and PS relate to cases where symptoms described include "tantrums", "mood of exultation", and "oppression". These words come from the vocabulary of mental disorders and may be familiar to MS from their training in psychiatry. However, some German investigators [10] administered questionnaires to different groups of students at different stages of their medical education. Among those just starting, attitudes towards pharmacotherapy in this context were broadly comparable with those seen in a general population. However, the similarity diminished as training progressed. It appeared that the more knowledge students acquired about psychiatry and pharmacology, the more favourable their attitudes became towards psychotropic drugs and other treatments (psychotherapy, etc.).

Although the difference was particularly pronounced in cases involving male adolescents, the available data do not allow the gender influence of the person described to be differentiated from that of other factors. One way to learn more about this, and to determine the content validity of the case reports, would be to repeat the survey having changed the forenames from male to female and *vice versa*.

Importantly, in only one case (Michel whose brother died 5 months previously) did the majority of both groups favour prescription. They considered Michel to be going through a period of mourning that was exhausting him and putting him at risk of suicide.

This study demonstrates that certain aspects of personality influence the advice a person gives concerning psychotropic medication. The findings here refer only to students, but they raise the question of whether personality traits have a similar influence on attitudes among practicing health professionals.

Interpretation of the NEO PI-R personality inventory data [20] indicates that those MS who have a tendency to feel anger, frustration and bitterness, and who show no misgivings in fulfilling moral duties would be more likely to prescribe psychotropic medication. Would that not be a quick and effective way to respond to a person in need?

PS who had difficulty facing up to stress, but were not shy, who were not unfriendly but showed interest in the well-being of others and had difficulty with social contact and tended not to think before acting would prescribe more psychotropic drugs. Someone quite vulnerable to stress himself and wanting to help others, might, without feeling an ounce of human warmth towards his peers, see prescribing psychotropic drugs as the most appropriate solution.

Limitations to be borne in mind include the difference in the numbers of PS and MS who volunteered. PS controls may have had a special interest in psychotherapeutic approaches given that they would never have the opportunity to prescribe; however, they would act as advisers and healthcare providers. Only students present on campus on the day of the investigation and who volunteered to participate were enrolled; they may not be representative of all the students registered in the respective faculties. The lack of reference to the type of medication (anti-depressants, anxiolytics, etc.) was intentional. With hindsight, data concerning respondents' own history of adverse life events and their management would have been of interest. But for ethical and confidentiality reasons, the researchers did not want the professors who authorized the study (see acknowledgements) to be able to identify responses from students in their classes.

Health promotion is not simply a matter of educating those young people who take psychotropic drugs – health professionals must also question the criteria that inform their decisions. It is as important to investigate the attitudes of the future health professionals (advisers or prescribers) as it is to focus on consumer-related issues.

## Competing interests

The author(s) declare that they have no competing interests.

## Authors' contributions

MB and ES participated equally in conceiving and carrying out the study and had the same responsibility for writing the manuscript.

Both authors read and approved the final manuscript.
